# Transepithelial phototherapeutic keratectomy for treatment-resistant recurrent corneal erosion syndrome

**DOI:** 10.1007/s00417-024-06482-1

**Published:** 2024-04-15

**Authors:** Mukhtar Bizrah, Maheshver Shunmugam, Geoffrey Ching, Radhika P. Patel, Nizar Din, David T. C. Lin, Simon P. Holland

**Affiliations:** 1 Imperial College Healthcare NHS Trust, Western Eye Hospital, 153-173 Marylebone Road, London, NW1 5QH UK; 2 The Department of Ophthalmology and Visual Sciences, University of British Columbia, Vancouver, Canada; 3 Imperial College London, London, UK; 4 Pacific Laser Eye Centre, Vancouver, Canada

**Keywords:** Recurrent corneal erosion syndrome (RCES), Phototherapeutic keratectomy (PTK), Transepithelial PTK, Corneal erosion, Anterior basement membrane dystrophy (ABMD), Epithelial debridement

## Abstract

**Background:**

To evaluate the efficacy and safety of trans-epithelial phototherapeutic keratectomy (TE-PTK) as a treatment for recurrent corneal erosion syndrome (RCES) in patients with symptoms refractory to conventional treatments.

**Methods:**

All patients who received TE-PTK treatment for RCES had failed 3 or more conventional treatments and were reviewed, and if met criteria, approved by healthcare workers of the British Columbia public health authority (Medical Services Plan (MSP). A retrospective chart review and telephone survey were conducted at the Pacific Laser Eye Centre (PLEC). Exclusion criteria were ocular co-morbidities potentially affecting treatment efficacy.

**Results:**

This study included 593 eyes of 555 patients (46.2% male; 50.9 ± 14.2 years old) who underwent TE-PTK. The leading identified causes of RCES were trauma (45.7%) and anterior basement membrane dystrophy (44.2%). The most common pre-PTK interventions were ocular lubricants (90.9%), hypertonic solutions (77.9%), and bandage contact lenses (50.9%). Thirty-six eyes had undergone surgical interventions such as stromal puncture, epithelial debridement, or diamond burr polishing. Post-PTK, 78% of patients did not require any subsequent therapies and 20% required ongoing drops. Six patients (1.1%) reported no symptom improvement and required repeat TE-PTK for ongoing RCES symptoms after initial TE-PTK. All 6 eyes were successfully retreated with TE-PTK (average time to retreatment was 11.3 ± 14.9 months). There was no significant difference in best corrected visual acuity pre- vs. post-operatively. The mean post-operative follow-up was 60.5 months (range: 5–127 months).

**Conclusion:**

TE-PTK has a good efficacy and safety profile for treatment-resistant RCES. The third-party public health–reviewed nature of this study, the low recurrence rate of RCES, and the low PTK retreatment rate suggest that TE-PTK might be considered for wider use in the management of RCES.

**Supplementary Information:**

The online version contains supplementary material available at 10.1007/s00417-024-06482-1.


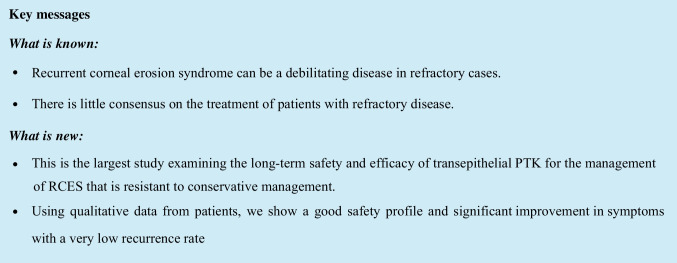


## Introduction

Recurrent corneal erosion syndrome (RCES) is a painful eye condition characterized by abnormal epithelial adhesion to underlying basal lamina. The most common etiology of RCES is mechanical trauma (45–64%), followed by anterior basement membrane dystrophy (ABMD) [1–3]. Untreated, RCES may lead to poor coupling of the corneal epithelium to the underlying basement membrane resulting in recurrent epithelial breakdown. RCES is detrimental to patient quality of life as it commonly has a significant impact on vision and presents with severe pain.

A conservative approach to RCES management involves measures such as the use of topical lubricants, topical hypertonic saline, and/or the use of a bandage contact lens [3]. More invasive approaches include mechanical corneal epithelial debridement, alcohol delamination of the epithelium, and excimer laser phototherapeutic keratectomy (PTK). A Cochrane review in 2018 which sought to explore the best treatment for RCES concluded that existing studies were of insufficient quality and size to guide treatment decisions [4]. When conservative measures fail, there is no uniform approach to the management of RCES.

To the best of our knowledge, this is currently the largest study examining the long-term safety and efficacy of transepithelial PTK for the management of RCES that is resistant to conservative management.

## Methods

### Study design

This study was a retrospective case series of 593 consecutive eyes from 555 patients with RCES that is resistant to conservative measures. All patients were treated at the Pacific Laser Eye Centre in Vancouver, British Columbia, Canada, and funded through the public healthcare system via the Special Authority Program (SAP) of the Medical Services plan of British Columbia. The treatment was publicly funded. Clinical notes were externally reviewed by physicians at the SAP, and funding was only granted for patients who failed three or more conservative treatments (see below). The study was approved by the Ethical Review Board of the Pacific Laser Eye Centre, Vancouver, British Columbia, Canada, and was conducted in adherence to the tenets of the Declaration of Helsinki.

The diagnosis of RCES was made based on a combination of the referral letter, the patient history of symptoms consistent with recurrent corneal erosions, and slit lamp examination findings, namely: epithelial erosions, corneal microcysts, negative fluorescein staining, map-dot-finger-print patterns. Patients were considered resistant to standard conservative treatment if they had tried 3 or more of the following:

1. Topical lubricants for $\ge$ 3 months
2. Topical hypertonic saline for $\ge$ 3 months
3. Topical steroid eye drops for $\ge$ 3 months
4. Oral doxycycline for $\ge$ 3 months
5. Bandage contact lens for $\ge$ 2 weeks

The inclusion and exclusion criteria for this study are shown below:

## Inclusion criteria:

1. Eyes with a history of RCES which have failed conservative treatment
2. Eyes with PTK treatment for RCES between January 12, 2010, and March 13, 2020.
3. Patients approved for publicly funded treatment through the Special Authority Program (SAP) of the Medical Services Program of British Columbia

## Exclusion criteria:

1. Eyes with band keratopathy
2. Eyes with significant scarring of the cornea secondary to previous corneal ulceration
3. Eyes with stromal dystrophy (e.g., granular, lattice, Avellino dystrophy)
4. Eyes with Reis-Bücklers corneal dystrophy

### Data collection

A retrospective case notes review was completed for all patients who underwent PTK for RCES between January 12, 2010, and March 13, 2020. To gather additional data about patient satisfaction, a random subset of 120 patients received a telephone survey.

Patient demographics, past ocular history, and PTK treatment outcome data were collected from chart review. Appendix 1 contains a questionnaire completed based on information in patient charts.

Appendix 2 contains a list of uniform standardized questions used in a telephonic survey completed by a subset of 120 patients. Information on patient symptom severity before and after PTK, therapies attempted pre-PTK, therapies needed post-PTK, and whether a patient would have PTK again was collected.

### Pre-operative assessment

Corrected distance visual acuity (CDVA) was measured with a phoropter and Snellen chart before treatment. Topography and tomography (SCHWIND Sirius, SCHWIND eye-tech solutions GmbH) were performed over a 12 mm in width.

### Surgical procedure

The SCHWIND Custom Ablation Manager (SCHWIND eye-tech solutions GmbH, Kleinostheim, Germany) set in transepithelial PTK mode. Sirius (C.S.O Frienze, Italy) combines Placido-disk topography with Scheimpflug tomography of the cornea. Sirius provides information on pachymetry, elevation, curvature, and dioptric power of both corneal surfaces over a diameter of 12 mm. All biometric measurements of the anterior chamber were calculated using 25 sections from the cornea. The devices used in this study meet the standards of European conformity (Conformité Européene or CE marking).

TE-PTK treatment protocol was conducted ablating 50 microns for epithelial removal and 15 microns of subepithelial treatment (total 65 microns). Topical anesthetics were instilled, and lid speculum was inserted. Alignment and positioning with the laser were achieved with a 1050 Hz infrared eye tracker with simultaneous limbus, pupil, and torsional tracking integrated into the laser system centered on the corneal vertex [5–7]. The eye tracker had a response time of 1.7 ms with a system total latency time of 2.9 ms. The ablation profile was centered on the corneal vertex determined by the topography using 100% of the pupil offset value, which closely approximates the visual axis [5–7]. Total treatment zone including ablation zone and transition zone was 9 mm. The excimer laser was operated in its transepithelial mode with a correction factor of + 0.50 D for 65 μm and above to avoid hypermetropic shift. This calibration factor was not used for patients who were myopic. Patients were requested to look at a pulsing green fixation light throughout the ablation. Mitomycin C 0.02% was applied for 30 s at the conclusion of the ablation followed by thorough ocular surface wash with balanced salt solution for 30 s. All patients were fitted with a therapeutic bandage contact lens (Acuvue® 8.4 mm or 8.8 mm). Patients were prescribed the following eye drops: Ciprofloxacin 0.3% three times per day for the duration of contact lens use, diclofenac 0.1% twice a day for 2 days, and fluorometholone 0.1% three times a day for a week, twice a day for a week, and then once a day for a week. Preservative-free lubricating eye drops were prescribed to be taken a minimum of 4 times daily.

For eyes requiring retreatment, the same aforementioned protocol was used for transepithelial PTK.

### Follow-up

Standard post-operative follow-up was arranged at 1 week, 2 weeks, 1 month, 3 months, 6 months, and annually thereafter. Most patients were followed up after the 1 week follow-up visit at their local optometrist/ophthalmologist office, and notes were sent to PLEC about their post-operative progress. These notes were kept in the patients’ clinical records.

At the initial 1 week follow-up, the bandage CL was removed and fluorescein eye drops were applied to the eye. If there was no cornea epithelial defect, then bandage CL was no longer used, and the patient followed up as per schedule above. If there was evidence of a persisting cornea epithelial defect, a bandage CL was re-applied. The patient was then followed up in 4–5 days either in the same clinic where the PTK laser treatment was conducted or at their local optometrist or ophthalmologist office. The bandage CL was removed again at this second visit, fluorescein eye drops applied, and cornea checked for any persistent epithelial defect. A bandage CL was no longer used once the epithelial defect healed.

### Safety

The clinical notes were reviewed for loss of visual acuity, development of cornea haze (grade 2 or above), and treatment failure.

### Statistical analysis

Demographic data, RCES characteristics, and telephone survey data were analyzed using descriptive statistics.

Data was recorded in Microsoft Excel (2019). The results are reported as mean ± standard deviation (SD) for patient’s age and follow-up time and as mean ± standard error of mean for all other measurements. Visual acuity was measured as logarithm of the minimum angle of resolution (logMAR) values for data analysis. Pre- and post-operative CDVA was analyzed by Student’s *t*-test.

The rate of recurrence was also calculated in eye-years, as the number of recurrences divided by the years of follow-up. Patients who complained of symptoms every day or every other day, without a recurrence-free interval of 3 months were counted as single recurrence. The Kaplan–Meier survival analysis was done to calculate the cumulative recurrence-free survival after treatment.

## Results

This study included 593 eyes of 555 patients who underwent TE-PTK for RCES between January 12, 2010, and March 13, 2020. Two hundred and seventy-four (46.2%) participants were male and three hundred and nineteen (53.8%) were female. Mean age of patients was 50.9 ± 14.2 years (Mean ± SD). The mean post-operative follow-up was 60.5 months (range: 5–127 months) post-PTK. Eighty-six percent of eyes had more than 18 months of follow-up, and 92% of eyes had a minimum of 12 months follow-up.

### RCES causes

Case notes review of pre-operative notes of 593 eyes showed that two hundred and seventy-one (45.7%) eyes had a history of corneal trauma while 262 (44.2%) eyes had a history of ABMD diagnosis. Sixty (10.1%) eyes did not have an identifiable cause of their RCES (Table 1). Thirty-four eyes (5.7%) had corneal scarring at initial visit.

**Table 1:** Patient RCES characteristics including etiology, symptoms, and interventions prior to PTK

	*N *(number of patients)	% (percentage)
RCES etiology		
Trauma	271	45.70
ABMD	262	44.20
Idiopathic	60	10.10
Symptoms		
Pain	389	65.60
Reduced vision	173	29.20
Photosensitivity	72	12.10
Foreign body sensation	252	42.50
Redness	61	10.30
Tearing	59	9.90
Previous interventions		
Lubricants	539	90.90
Hypertonics	462	77.90
Antibiotics	30	5.10
Topical NSAIDs	122	20.60
MMP inhibitors	10	1.70
Steroids	90	15.20
Bandage contact lens	302	50.90
Anterior stromal micro-puncture	15	2.50
Diamond burr polishing	1	0.20
Epithelial debridement	15	2.50
Other	2	0.30

### Symptoms

Eye pain was the chief complaint of 389 (65.6%) of 593 eyes at initial consultation (Table 1). Foreign body sensation was also present in 252 (42.5%) eyes, while reduced vision was noted in 173 (29.2%) eyes. Photosensitivity (12.1%), red eye (10.3%), and tearing (9.9%) were other reported complaints at initial patient consult.

### Prior treatments

Conservative treatments included the use of ocular lubricants, which were the most frequently used pre-PTK medical intervention, as seen in 539 (90.9%) of 593 eyes (Table 1). Hypertonic solutions (77.9%), bandage contact lenses (50.9%), and topical NSAIDs (20.6%) were used in a large proportion of eyes prior to PTK. Steroids eye drops (15.2%), antibiotic eye drops (5.1%), and oral doxycycline (1.7%) were used less commonly. All eyes had at least three conservative interventions tried for a minimum of 3 months.

A minority of eyes underwent surgical interventions prior to PTK. Fifteen eyes (2.5%) had undergone anterior stromal puncture; 15 eyes (2.5%) had undergone an epithelial debridement. One eye (0.17%) underwent diamond burr polishing. Two (0.34%) eyes had undergone other, unknown surgical interventions prior to PTK.

### Patient telephone survey

One hundred and twenty (20.2%) patients completed a post-PTK satisfaction assessment via telephonic survey. On average, surveys were completed 5 years post-PTK (Table 2).

**Table 2:** Telephone survey data from 112 RCES patients who were asked about their symptoms post-PTK compared to before, whether they would have PTK again, and whether they received any further treatment after PTK

	*N *(number of patients)	% (percentage)
Symptom control		
Worse	1	0.9
Same	5	4.5
Better	56	50.0
Completely resolved	50	44.6
Would have PTK again		
Yes	104	93
No	8	7
Received subsequent treatment		
No	87	78
Medical	22	20
Surgical	3	3

Ninety-one (75.8%) survey participants did not require any ongoing RCES treatment post-PTK. Twenty-four (20%) respondents continued to use conservative therapy post-PTK including lubricants (100%), hypertonic solution (16.7%), and bandage contact lens (4.2%). Five (4.2%) respondents underwent a second surgical therapy post-PTK for RCES symptoms.

One hundred and four (86.7%) patients would choose PTK for treatment-resistant RCES again; however, sixteen (13.3%) would not choose the procedure again. Of those who would not choose PTK again, four (25%) required a second surgical treatment and nine (56.3%) required ongoing conservative treatments.

Forty-eight (44.4%) patients endorsed complete symptom resolution, while 56 (51.9%) claimed their symptoms had decreased. Four (3.7%) patients reported no symptomatic change post-PTK, and 1 (0.9%) patient reported worsening symptoms.

### Retreatment

Six (1%) eyes required repeat PTK for ongoing RCES symptoms after initial PTK. The average time to retreatment was 11.3 ± 14.9 months. Fifty percent of these eyes had underlying ABMD and 50% had prior corneal trauma. All six eyes were successfully retreated with PTK and were followed up for 1 year. They did not require further treatment thereafter. No other eyes required surgical retreatment in the form of PTK or other invasive intervention.

### Visual acuity

Pre-operative and post-operative CDVA data were collected from a randomly generated sample of 126 eyes that underwent PTK for RCES. Mean CDVA (logMAR) improved from 0.15 ± 0.27 pre-operatively to 0.11 ± 0.17 post-operatively (*p* = 0.16) shown. The improvement in visual acuity was not statistically significant.

**Fig. 1:**
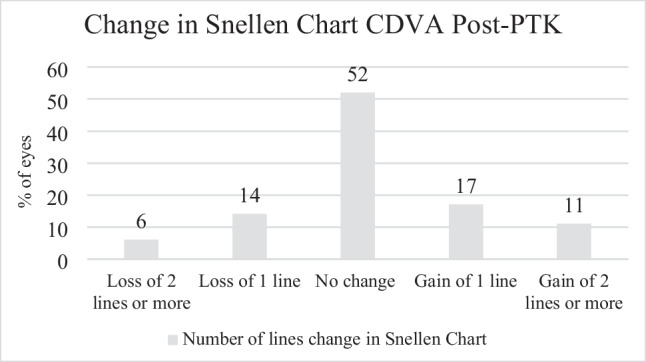
Change in corrected visual acuity recorded via Snellen chart post-PTK

Fifty-two percent of patients had no change in CDVA. Twenty-eight percent of patients had an improved CDVA post-operatively with an average of 2 lines improvement on the Snellen chart. Twenty percent of patients experienced worse CDVA with an average decrease of 1.5 Snellen lines. CDVA results are depicted in Fig. 1.

### Safety

Twenty percent of patients experienced a decrease in CDVA; however, no statistical difference existed between pre- and post-operative CDVA. The reasons for visual loss are displayed in Table 3. The average loss of lines on a Snellen chart was 1.5 among 20% of patients who experienced any loss in CDVA. Nine (1.6%) of 593 eyes had grade 2 cornea haze, and no eyes had grade 3 or more cornea haze.

**Table 3:** Patients with > 2 lines of CDVA lost with reasons for CDVA loss

Patient number	Lines of CDVA Lost	Reason for CDVA Loss
Patient #1	2	Significant diffuse superficial punctate keratopathy. The eye was treated with *Xiidra* (lifitegrast ophthalmic solution) 5% and Muro 128 (hypertonic sodium chloride eye ointment) resulting in vision improvement to 20/40
Patient #2	4	Fuchs endothelial dystrophy with cataract
Patient #3	2	Subepithelial cornea scarring
Patient #4	3	Persistent cornea epithelial defect. Infective microbial keratitis suspected. Treated with ciprofloxacin eye drops. Residual cornea scarring
Patient #5	3	Corneal scarring post pterygium excision
Patient #6	3	Previous cornea subepithelial opacities. Possible worsening of subepithelial scarring

## Discussion

This study illustrates the benefits of transepithelial PTK for eyes with RCES that have failed multiple conservative treatments. The benefits of transepithelial PTK illustrated in our study include high patient satisfaction (87%), a high proportion no longer requiring conservative treatment (80%), no statistically significant change in CDVA, low risk of cornea haze (grade 2 cornea haze, 1.4%), limited need for invasive retreatment (1%), and improvement or resolution of symptoms (96%). As, we believe PTK may be one of the most effective treatment options for such eyes. PTK requires a laser suite, surgical experience, and may be more expensive than other invasive treatments.

Our study found that 44% of eyes with treatment-resistant RCES had an underlying diagnosis of ABMD in comparison to the literature which reports a rate of 26–29% [1–3]. One explanation may be that the likelihood of developing treatment-resistant RCES is higher in those patients with an underlying diagnosis of ABMD. [8] The cohort of patients included in this study were exclusively treatment resistant and therefore represent a unique subset of patients with RCES.

Globally, 50–60% of patients with RCES require invasive intervention as they remain symptomatic despite conservative management [3, 9]. Several invasive treatments for RCES include anterior stromal micro-puncture, alcohol delamination of the epithelium, mechanical corneal epithelial debridement, and diamond burr polishing exist; however, no robust evidence suggests one invasive treatment is superior to another [4].

The potential advantages of anterior stromal micro-puncture in comparison to PTK include decreased cost and the ability to complete this procedure in an outpatient clinic [10]. Disadvantages include a higher percentage of patients requiring a second treatment (17–24%) in comparison to PTK (6%) [10]. Additionally, faint corneal scars are nearly always present secondary to treatment which are not usually visually significant. Epithelial map–guided anterior stromal micro-puncture may have an improved treatment safety and efficacy for RCES; however, large trials are needed to establish reliable outcome [11]. The evidence on stromal micro-puncture is limited which limits the ability to compare efficacy and safety between stromal micro-puncture and PTK. In our study, only 1% of eyes required a repeat surgical treatment.

In a randomized controlled trial of 17 eyes treated with alcohol delamination of the corneal epithelium (ADCE) and 16 eyes treated with PTK, there was complete or partial resolution of symptoms in 65% of eyes in the ADCE group and 63% of eyes in the PTK group [12]. Mean follow-up was 16.25 and 17.25 months, respectively. Recurrence of symptoms occurred in 5 eyes in the ADCE group and 6 eyes in the PTK group, with no long-term complications in either group. In another study of 26 eyes followed for a minimum of 3 years, 11.5% of eyes that underwent ADCE had recurrence [13]. This study was limited by the small sample size. Additionally, no eyes in this study had an underlying diagnosis of ABMD. In our study, 96% of patients achieved complete resolution or improvement of symptoms after PTK and 87% of patients would undergo PTK for treatment-resistant RCES again based on post-PTK telephonic interview.

There is evidence to suggest epithelial debridement and diamond burr polishing may result in resolution of RCES symptoms in up to 88–97% of cases [14, 15]; however, disadvantages of this technique include the risk of developing post-operative corneal haze (14–26%) [14–16]. In comparison, only 1.6% of eyes in our study developed grade 2 cornea haze, and none developed grade 3 or more.

In this study, six patients (1%) required RCES retreatment with PTK and 20% of patients required ongoing conservative treatment post-PTK. No significant change was noted between mean pre-operative and mean post-operative CDVA; however, nearly a third of patients (28%) experienced an improved CDVA. There is evidence to suggest that surface treatments have potential to improve vision by treating epithelial irregularity and associated irregular astigmatism, particularly in ABMD [15, 17]. Further research should be done to examine the astigmatism outcomes of patients who undergo PTK for RCES.

Unlike PTK, alcohol delamination of the corneal epithelium, manual epithelial debridement, and diamond burr polishing do not include ablation of the corneal stroma. For this reason, these invasive procedures have zero risk of inducing a hyperopic shift.

In myopic eyes, the hyperopic shift may be a desirable side effect. In emmetropic and hypermetropic eyes, this shift may result in blurring of UDVA and subsequent patient dissatisfaction. In this study, stromal ablation was limited to 10 microns, and treatment diameter was 9 mm, to minimize the risk of hyperopic shift. The excimer laser was operated in transepithelial mode, and a correction factor of + 0.50 D for 65 μm was used to achieve a neutral refractive outcome. This calibration factor was not used for myopic eyes.

Corneal haze is a known complication of PTK, ADCE, mechanical epithelial debridement, and diamond burr polishing. For this reason, eyes undergoing these treatments are commonly treated with a post-operative course of steroids and the use of a bandage contact lens to reduce the risk of corneal haze. It is important to note that use of steroid eye drops and a bandage contact lens are themselves known treatment options for RCES, which may affect treatment outcomes. In our study, only 1.6% of eyes in our study developed grade 2 cornea haze, and none developed grade 3 or more. This is lower than other studies reporting PTK treatment outcomes for RCES [18]. This difference may be due to the use of Mitomycin C 0.02% (MMC) which is used on all eyes for 30 s following corneal ablation to minimize the risk of cornea haze development. Due to concerns about MMC cytotoxic effects, there is large variability in its usage for small refractive errors and standard PTK treatment; however, there were no reported MMC complications in our study.

The strengths of this study include the large sample size, long follow-up, and additional third-party vetting of treatment-resistant RCES by MSP for publicly funded PTK. Limitations of this study include its retrospective nature, no data collection on uncorrected distance visual acuity (UDVA), only a sample (124 eyes) collected on change in CDVA, focus of the study on recurring RCES symptoms, no control group, post-operative refractive data, and non-full patient participation in post-PTK telephonic interviews. This data was not available as TE-PTK in our study was performed for the sole purpose of decreasing RCES symptoms; therefore, this data was not fully available to include in analysis.

## Conclusion

Our study represents the largest analysis of eyes that underwent transepithelial phototherapeutic keratectomy (TE-PTK) for treatment-resistant recurrent corneal erosion syndrome (RCES) as far as we are aware. The public health pre-operative review of eligibility, the high patient satisfaction, and the low recurrence rate of RCES indicate that TE-PTK is safe and effective. We encourage further studies to look into benefits of PTK in earlier stages of RCES management.

## Supplementary Information

Below is the link to the electronic supplementary material.

Supplementary file1 (XLSX 33 KB)

Supplementary file2 (DOCX 20 KB)

## References

[1] Hykin PG, Foss AE, Pavesio C, Dart JK (1994) The natural history and management of recurrent corneal erosion: a prospective randomised trial. Eye (Lond) 8(Pt 1):35–40. 10.1038/eye.1994.6 8013716 10.1038/eye.1994.6

[2] Reeves SW, Kang PC, Zlogar DF, Gupta PK, Stinnett S, Afshari NA (2010) Recurrent corneal erosion syndrome: a study of 364 episodes. Ophthalmic Surg Lasers Imaging 1–2 10.3928/15428877-20100215-44 10.3928/15428877-20100215-44 20337327

[3] Reidy JJ, Paulus MP, Gona S (2000) Recurrent erosions of the cornea: epidemiology and treatment. Cornea 19:767–771. 10.1097/00003226-200011000-00001 11095047 10.1097/00003226-200011000-00001

[4] Watson SL, Leung V (2018) Interventions for recurrent corneal erosions. Cochrane Database Syst Rev 7:CD001861. 10.1002/14651858.CD001861.pub4 29985545 10.1002/14651858.CD001861.pub4 PMC6513638

[5] Arba Mosquera S, Ewering T (2012) New asymmetric centration strategy combining pupil and corneal vertex information for ablation procedures in refractive surgery: theoretical background. J Refract Surg 28:567–573. 10.3928/1081597X-20120703-01 22785059 10.3928/1081597X-20120703-01

[6] Arbelaez MC, Vidal C, Arba-Mosquera S (2008) Clinical outcomes of corneal vertex versus central pupil references with aberration-free ablation strategies and LASIK. Invest Ophthalmol Vis Sci 49:5287–5294. 10.1167/iovs.08-2176 18658090 10.1167/iovs.08-2176

[7] De Ortueta D, ArbaMosquera S (2007) Centration during hyperopic LASIK using the coaxial light reflex. J Refract Surg 23:11. 10.3928/1081-597X-20070101-02. (**author reply 11**) 17269237 10.3928/1081-597X-20070101-02

[8] Dedes W, Faes L, Schipper I, Bachmann LM, Thiel MA (2015) Phototherapeutic keratectomy (PTK) for treatment of recurrent corneal erosion: correlation between etiology and prognosis-prospective longitudinal study. Graefes Arch Clin Exp Ophthalmol 253:1745–1749. 10.1007/s00417-015-2990-6 25900814 10.1007/s00417-015-2990-6

[9] Heyworth P, Morlet N, Rayner S, Hykin P, Dart J (1998) Natural history of recurrent erosion syndrome–a 4 year review of 117 patients. Br J Ophthalmol 82:26–28. 10.1136/bjo.82.1.26 9536875 10.1136/bjo.82.1.26 PMC1722350

[10] McLean EN, MacRae SM, Rich LF (1986) Recurrent erosion. Treatment by anterior stromal puncture. Ophthalmology 93:784–788 3737123

[11] Oikonomakis K, Petrelli M, Petrovic A, Andreanos K, Droutsas K, Georgalas I, Kymionis G (2019) Epithelial map-guided anterior stromal micropuncture for the treatment of recurrent corneal erosion syndrome. Int Ophthalmol 39:943–948. 10.1007/s10792-018-0891-5 29557084 10.1007/s10792-018-0891-5

[12] Chan E, Jhanji V, Constantinou M, Amiel H, Snibson GR, Vajpayee RB (2014) A randomised controlled trial of alcohol delamination and phototherapeutic keratectomy for the treatment of recurrent corneal erosion syndrome. Br J Ophthalmol 98:166–171. 10.1136/bjophthalmol-2013-303276 23759442 10.1136/bjophthalmol-2013-303276

[13] Teh BL, Chua PYS, Reddy AR (2021) Three-year outcomes of alcohol delamination of corneal epithelium for recurrent corneal erosions of traumatic etiology. Indian J Ophthalmol 69:2437–2440. 10.4103/ijo.IJO_3796_20 34427239 10.4103/ijo.IJO_3796_20 PMC8544076

[14] Sridhar MS, Rapuano CJ, Cosar CB, Cohen EJ, Laibson PR (2002) Phototherapeutic keratectomy versus diamond burr polishing of Bowman’s membrane in the treatment of recurrent corneal erosions associated with anterior basement membrane dystrophy. Ophthalmology 109:674–679. 10.1016/s0161-6420(01)01027-2 11927423 10.1016/s0161-6420(01)01027-2

[15] Vo RC, Chen JL, Sanchez PJ, Yu F, Aldave AJ (2015) Long-term outcomes of epithelial debridement and diamond burr polishing for corneal epithelial irregularity and recurrent corneal erosion. Cornea 34:1259–1265. 10.1097/ICO.0000000000000554 26203746 10.1097/ICO.0000000000000554

[16] Itty S, Hamilton SS, Baratz KH, Diehl NN, Maguire LJ (2007) Outcomes of epithelial debridement for anterior basement membrane dystrophy. Am J Ophthalmol 144:217–221. 10.1016/j.ajo.2007.04.024 17553446 10.1016/j.ajo.2007.04.024

[17] Zalentein WN, Holopainen JM, Tervo TM (2007) Phototherapeutic keratectomy for epithelial irregular astigmatism: an emphasis on map-dot-fingerprint degeneration. J Refract Surg 23:50–57. 10.3928/1081-597X-20070101-09 17269244 10.3928/1081-597X-20070101-09

[18] Miller DD, Hasan SA, Simmons NL, Stewart MW (2019) Recurrent corneal erosion: a comprehensive review. Clin Ophthalmol 13:325–335. 10.2147/OPTH.S157430 30809089 10.2147/OPTH.S157430 PMC6376883

