# Supplementary material for: Transepithelial phototherapeutic keratectomy for treatment-resistant recurrent corneal erosion syndrome
Source: Graefes Arch Clin Exp Ophthalmol. 2024 Apr 15;262(10):3253–60. doi: 10.1007/s00417-024-06482-1 (PMC11458720; doi:10.1007/s00417-024-06482-1)
Supplement: Supplementary file 2 — Supplementary file2 (DOCX 20 KB) [file 417_2024_6482_MOESM2_ESM.docx]

RCES Questionnaire

**“By completing the questionnaire, you are consenting to participate in this research.”**

**“If you have any concerns or complaints about your rights as a research participant and/or your experiences while participating in this study, contact the Research Participant Complaint Line in the UBC Office of Research Ethics at 604-822-8598 or if long distance e-mail RSIL@ors.ubc.ca or call toll free 1-877-822-8598. “Please reference the study number [Hxx-xxxxx] when calling so the Complaint Line staff can better assist you.”**

"What symptoms did you experience that led you to seek treatment for your corneal erosion?"

"What do you believe caused your recurrent corneal erosions?"

"How well are your symptoms currently controlled?" **SEE BELOW**

"What treatments had not worked before you had the laser treatment?"

"Was any other treatment by a doctor needed after laser?"

"Would you have PTK again?"

DEFINITIONS USED FOR SYMPTOMS SEVERITY:

WORSE: RCES SYMPTOMS WORSENED AFTER PTK

SAME: NO CHANGE IN RCES SYMPTOMS

BETTER: RCES SYMPTOMS IMPROVED BUT DID NOT COMPLETELY RESOLVE

COMPLETELY RESOLVED: NO MORE RCES SYMPTOMS AFTER PTK TREATMENT.
